# setsApp for Cytoscape: Set operations for Cytoscape Nodes and Edges

**DOI:** 10.12688/f1000research.4392.2

**Published:** 2015-08-05

**Authors:** John H. Morris, Samad Lotia, Allan Wu, Nadezhda T. Doncheva, Mario Albrecht, Alexander R. Pico, Thomas E Ferrin

**Affiliations:** 1 Resource for Biocomputing, Visualization, and Informatics, University of California, San Francisco, CA 94143, USA; 2 Gladstone Institutes, San Francisco, CA, USA; 3 Max Planck Institute for Informatics, Saarbrücken, 66123, Germany; 4 University Medicine Greifswald, Greifswald, 17489, Germany; 5 Graz University of Technology, Graz, 8010, Austria; 6 BioTechMed-Graz, Graz, 8036, Austria

**Keywords:** cytoscape, app, sets functionality

## Abstract

setsApp ( http://apps.cytoscape.org/apps/setsapp) is a relatively simple Cytoscape 3 app for users to handle groups of nodes and/or edges. It supports several important biological workflows and enables various set operations. setsApp provides basic tools to create sets of nodes or edges, import or export sets, and perform standard set operations (union, difference, intersection) on those sets. Automatic set partitioning and layout functions are also provided. The sets functionality is also exposed to users and app developers in the form of a set of commands that can be used for scripting purposes or integrated in other Cytoscape apps.

## Introduction

Cytoscape ^1, 2^ provides an environment for the visualization and analysis of networks and associated annotations. The primary audience for Cytoscape is the biological community and Cytoscape supports a number of standard use cases for analyzing and visualizing biological data. Many of these use cases involve the selection of a number of nodes or edges based on some analysis or annotation and either performing an action on that selection or comparing those nodes or edges to a different set of nodes or edges that resulted from alternative analyses or analyses based on alternative annotations. The core capabilities for Cytoscape provide some tools to facilitate these types of comparisons but they can be counterintuitive or complicated to use. *setsApp* is a Cytoscape 3 application that provides a general set of tools for users and developers to define and maintain sets of nodes or edges and compare those sets using the standard set operations of union, intersection, and difference. Partition and layout features are also provided to assist in generating sets and performing set-aware layouts.

In this paper, we present the implementation of *setsApp*, in particular, how *setsApp* integrates with the Cytoscape command system, and then present a sample biological workflow using *setsApp*.

## Implementation

Cytoscape provides two approaches to implementing apps: a simple app and a bundle app. Simple apps are implemented using the same general approach as in Cytoscape 2.8, but at the cost of significant flexibility. Bundle apps utilize Open Service Gateway Initiative (OSGi) ^3^ interfaces through APIs provided by Cytoscape to interact with the Cytoscape core functionality. *setsApp* is implemented as a bundle app and utilizes the Cytoscape 3.1.0 API. There are three main components to the *setsApp* implementation: the user interface, the command interface, and the underlying data model for maintaining sets of nodes and edges.

### User interface

The *setsApp* user interface consists of menu items in the main Apps menu, node and edge context menus, and a panel added to the **Control Panel** (left or west) section of the Cytoscape user interface. The main feature of the **Sets** panel is the list of currently defined sets. Each set can be expanded to see all of the nodes or edges within that set, and context menus provide the ability to select, deselect, rename, or remove sets. As long as you have one set defined, **Partition** and **Layout** buttons will be active at the bottom of the panel. The partition feature will create new sets based on shared and excluded sets present in all currently defined sets. The layout feature will perform the selected layout algorithm taking into account set memberships, i.e., attempting to keep sets close together. When multiple sets are selected, the **Set Operations** buttons are enabled. This allows users to create new sets based on the union, intersection, or difference of other sets. Note that the results of a union or intersection are well-defined for multiple sets, but the difference operation is order dependent. If only two sets are selected, the order of selection is preserved. If more than two sets are selected, the order is the order of selection, so care must be taken when attempting to create a difference set of more than two sets. Sets can be created from the currently selected nodes or edges, or based on a particular node or edge attribute. When creating sets from attributes, the user will need to supply a prefix for the sets to be created and choose the attribute (currently only String attributes are supported) from a list. Sets can also be created by importing them from a simple text file. Each set can be individually exported to a text file.

*setsApp* provides a context menu for sets and set members in the control pane. In addition to having menu items to manage sets, the user may select all set members in the network, or if the set is expanded, individual members. This functionality presents an easy way for users to visualize the results of set operations and to perform interactive exploratory analysis.

The menus provided through the top-level Apps menu offer the same functionality as the **Create set from** menu in the *setsApp* control panel with the addition of a menu to import a set from a file. Node and edge context menus provide the user with the ability to add or remove the corresponding node or edge from sets.

### Command interface

In addition to the standard user interface described above, *setsApp* provides a number of “commands”. These commands may be used for scripting purposes or by other Cytoscape apps that wish to take advantage of the *setsApp* functionality. Table 1 provides a list of commands and the arguments.

**Table 1.** *setsApp* Commands. Arguments with asterisks are required.

Command	Arguments	Description
*setsApp* addTo	**edgeList**= list of edges* **nodeList**= list of nodes* **network**= *network to use* **name**= name of set*	Adds the listed nodes or edges to the named set. An error will occur if the types (node or edge) do not match or if the set does not exist. The set name and one of edgeList or nodeList are required.
*setsApp* createSet	**edgeList**= list of edges* **nodeList**= list of nodes* **network**= *network to use* **name**= name of set*	Creates the set from the edge or node list. It throws an error if both edge and node lists are provided.
*setsApp* difference	**name**= name of new set* **set1**= name of the first set* **set2**= name of the second set*	Performs a difference of two sets and puts the result into a new set.
*setsApp* export	**Column**= *column containing the* *id key* **name**= name of set* **setFile**= path to the file to import*	Exports a set to the specified file using the designated column to identify the node or edge.
*setsApp* import	**Column**= *column containing the* *id key* **Type**= [Node|Edge]* **name**= name of set* **setFile**= path to the file to import*	Exports a set to the specified file using the designated column to match the node or edge identifier from the file.
*setsApp* intersect	**name**= name of new set* **set1**= name of the first set* **set2**= name of the second set*	Performs an intersection of two sets and puts the result into a new set.
*setsApp* remove	**name**= name of set*	Removes (deletes) the set.
*setsApp* removeFrom	**edgeList**= list of edges* **nodeList**= list of nodes* **network**= *network to use* **name**= name of set*	Removes the listed nodes or edges from the named set. An error will occur if the types (node or edge) do not match or if the set does not exist. The set name and one of edgeList or nodeList are required.
*setsApp* rename	**newName**= *new name for the set* **oldName**= *old (current) name for* *the set*	Renames an existing set.
*setsApp* union	**name**= name of new set* **set1**= name of the first set* **set2**= name of the second set*	Performs a union of two sets and puts the result into a new set.

A command is made available to Cytoscape by creating a standard Cytoscape TaskFactory with two new properties in the org.cytoscape.work package: ServiceProperties.COMMAND_NAMESPACE, which is always set to “ *setsApp*”; and ServiceProperties.COMMAND, which is the command name (e.g. “addTo”). The command arguments are implemented as Tunables within the Task called by the designated TaskFactory. Because there is no guarantee that the Task will be executed within the context of a GUI, care should be taken to make sure that the appropriate Tunable types are used. For example, the NodeList Tunable allows the command-line user to enter a list of nodes rather than assuming that the user will have selected nodes interactively.

For another Cytoscape app to use any of these commands, it would need to call one of the Cytoscape TaskManagers and provide it org.cytoscape.command.CommandExecutorTaskFactory’s createTaskIterator method with the appropriate argument map, command, and command namespace. The TaskObserver method may be used if the command returns any values. For example:

**Listing 1. Example Command Usage.**



                        SynchronousTaskManager tm =
  serviceRegistrar. getService
      (SynchronousTaskManager.
                        class
                        );
CommandExecutorTaskFactory cetf =
  serviceRegistrar. getService
      (CommandExecutorTaskFactory.
                        class
                        );
Map<String, Object> argMap = 
                        new
    
                        HashMap<String, Object>();
argMap.put(
                        "name"
                        , 
                        "NewSet"
                        );

                        // selected is a special keyword for the
    
                        NodeList tunable

                        argMap.put(
                        "nodeList"
                        ,
                        "selected"
                        );

                        // the current network

                        argMap.put(
                        "network"
                        ,
                        "current"
                        );

                        // Assumes that this implements TaskObserver

                        tm.execute(cetf.createTaskIterator(
                        "setsApp"
                        ,
    
                        "createSet"
                        , 
                        argMap, 
                        this
                        ), 
                        this
                        );
                    


### Data model

The main model object for a node or edge set is the *Set* object, which stores a map of all of the nodes or edges in this set. A *SetsManager* provides the methods to create and destroy sets. The *SetsManager* also serves the critical function of serializing the information about Sets to the default hidden table (see CyNetwork.HIDDEN_ATTRS) for nodes or edges (depending on the type of the Set). Each Set is created as a boolean column in the hidden table which is set to **true** if the corresponding node or edge is in that set. By storing values in the default hidden tables, the information about sets is automatically saved in Cytoscape sessions and restored when sessions are reloaded. *SetsManager* implements *SessionReloadedListener* and recreates the *Set*s from the information stored in the hidden table columns.

## Results

**Figure 1.**
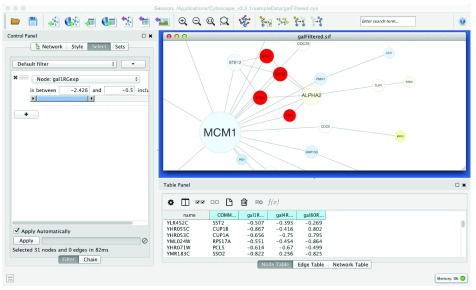
Screenshot of Cytoscape’s Select panel with underexpressed genes in the gal1RGexp condition being selected. Nodes that match the gal1RGexp condition are highlighted in red.

**Figure 2.**
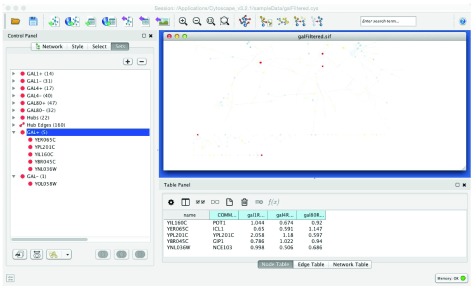
Screenshot of Cytoscape showing the Sets panel with all condition sets created. Nodes in the GAL+ set are highlighted in red.

A simple example use case might be the exploration of the data set from Ideker *et al.*, 2001 ^4^, which measured the change in expression for 331 genes after a systematic deletion of genes known to be involved in the *Saccharomyces cerevisiae* switch to galactose metabolism. This data was combined with known protein-protein and protein-DNA interactions to explore the biological response to deletions in the presence (the G in the column names) or absence of galactose in the medium. This data set is now included as a sample with Cytoscape downloads ( *galFiltered.cys*). In our workflow we use Cytoscape’s **Select** panel to select all proteins that are underexpressed (gal1RGexp < -0.5 fold change) in the deletion of GAL1 ( Figure 1). That selection is saved as a set named GAL1- ( **Apps→ *SetsApp*→Create node set**). We then select all of the proteins that are overexpressed (gal1RGexp > 0.5 fold change) in the deletion of GAL1 and save that selection as a set named GAL1+. Repeating this for GAL4 (column gal4RGexp) and GAL80 (column gal80Rexp) results in 6 sets altogether: GAL1+, GAL1-, GAL4+, GAL4-, GAL80+, GAL80- ( Figure 2). Note that the data for GAL1 and GAL4 is in the presence of galactose, but the data for GAL80 is in the absence of galactose since GAL80 is a known repressor of GAL4.

Given those six sets of genes, we can explore the data sets by looking at combinations of the sets. For example, we could look at the intersection of all of the underexpressed proteins by selecting each of GAL1-, GAL4-, and GAL80- in the **Sets** panel and pressing the **Intersection** button in the **Set Operations** box near the bottom of the panel. If we name the resulting set GAL- we see that it contains a single gene: YOL058W (ARG1). In this data set of 331 genes, only this one gene is repressed for all three of the deleted GAL genes. In the absence of galactose when GAL80 is deleted, ARG1 is underexpressed, and in the presence of galactose when either GAL1 or GAL4 are deleted the gene is also underexpressed. Looking at the expression significance values in the Node Table Panel of Cytoscape (gal1RGsig, gal4RGsig, and gal80Rsig) this is a highly significant result, although there is no direct correlation between the galactose switch and arginine biosynthesis regulation that we were able to find in the literature. On the other hand, ARG1 is regulated by the GCN4 activator which is known to repress protein synthesis during periods of stress or starvation ^5^, which explains the significant down-regulation of ARG1. We can perform a similar analysis to understand the consistent up-regulation of the five genes in the GAL+ set corresponding to gene symbols: GIP1, NCE103, YIG1, POT1, and ICL1. Figure 2 shows the results of the intersection operations.

We can also explore data sets by using layout algorithms that are informed by the sets we created earlier. By default, Cytoscape provides over a dozen layout algorithms that spatially place nodes in order to help elucidate meaningful patterns of relationships in networks. Most layout algorithms take into account connectivity between nodes. *SetsApp* augments Cytoscape by supplying two additional layout algorithms that take set membership into account. The setsbased grid layout places nodes in the same set together into independent grids, i.e., a grid of grids. This is ideal for quickly separating nodes in different sets for manual manipulation later on. The setsbased force directed layout employs the Prefuse force directed layout provided by Cytoscape but also tries to put nodes in the same set closer together in the network. Users can adjust the force between nodes in a given set relative to connected nodes and thereby emphasize or diminish the grouping based on set membership by changing values in the layout settings panel ( **Layout**→ **Settings**... ).

## Conclusions

There are many Cytoscape workflows that could take advantage of the features of *setsApp*. Any workflow that might want to look for groups of nodes or edges that share multiple traits, or that explicitly do not share those traits. While it is possible to duplicate many of the final results enabled by *setsApp* by using Cytoscape 3.1’s new **Select** panel, a user would need to know in advance exactly the combination of features that were biologically interesting. *setsApp* provides an alternative that allows users to explore various combinations of nodes and edges and to save such selections for later uses. We also provide layout algorithms that take set membership into consideration.

In the workflow we developed above, we combined the functionality of Cytoscape’s **Select** panel with *setsApp* to explore combinations of sets of genes based on shared properties. There are many more sophisticated apps available to Cytoscape users that could be used to do a more thorough analysis of this data set including jActiveModules ^6^, clusterMaker ^7^ and RINalyzer ^8^, however, the workflow above demonstrates the utility of a simple set-oriented approach to exploring networks.

## Software availability

Software available from: http://apps.cytoscape.org/apps/setsApp

Latest source code: https://github.com/RBVI/setsApp

Source code as at the time of publication: https://github.com/F1000Research/setsApp

Archived code as at the time of publication: http://www.dx.doi.org/10.5281/zenodo.10424 ^9^

License: Lesser GNU Public License 3.0: https://www.gnu.org/licenses/lgpl.html
