# Supplementary material for: setsApp for Cytoscape: Set operations for Cytoscape Nodes and Edges
Source: F1000Res. 2015 Aug 5;3:149. Originally published 2014 Jul 1. [Version 2] doi: 10.12688/f1000research.4392.2 (PMC4207244; doi:10.12688/f1000research.4392.2)
Supplement: Peer review history

# Peer review: Referee response for version 2

**DOI:** 10.5256/f1000research.7271.r9845

**Published:** 2015-08-06

**Authors:** Tamara Munzner

**Affiliations:** 1 Department of Computer Science, University of British Columbia, Vancouver, BC, Canada

Revisions are reasonable.

I have read this submission. I believe that I have an appropriate level of expertise to confirm that it is of an acceptable scientific standard.

# Peer review: Referee response for version 1

**DOI:** 10.5256/f1000research.4701.r5299

**Published:** 2014-07-28

**Authors:** Tamara Munzner

**Affiliations:** 1 Department of Computer Science, University of British Columbia, Vancouver, BC, Canada

This short article clearly describes the setsApp plugin for Cytoscape and walks the reader through example analysis. It ends with a useful disclaimer that the goal of the app is to provide users with a simple workflow, rather than completely novel capabilities.

The paragraph covering TaskFactory details will probably require multiple passes for readers unfamiliar with Cytoscape internals, but the major point can still be gleaned from the write-up as it stands.

Minor issue: 

It would be easier to read if the Figures were renumbered so that they match the order of discussion in the text; now Figure 1 comes after 2 and 3.

I have read this submission. I believe that I have an appropriate level of expertise to confirm that it is of an acceptable scientific standard.

# Peer review: Referee response for version 1

**DOI:** 10.5256/f1000research.4701.r5302

**Published:** 2014-07-14

**Authors:** Thomas Kelder

**Affiliations:** 1 EdgeLeap B.V., Utrecht, Netherlands

The authors describe setsApp for Cytoscape, an app that provides a user and developer friendly way to define sets of nodes and edges and perform union, intersect and difference operations on these sets. The features of this app are rather basic (set definition and simple set operations), but nevertheless provide a very useful extension to core Cytoscape functionality. By designing the app so that it can be used through either the GUI or the commands interface makes it a very powerful utility component that can be used in different use cases, workflows, or as building block in other apps. The manuscript is well written and provides a clear and detailed description of the app data model, usage instructions and use case. I tested the app using Cytoscape 3.1.1 and everything worked as described.

**Minor comments on the manuscript:** 

1. The paragraph following Table 1 is very technical. A code example to illustrate the use of Tunables would be useful. Also consider placing this paragraph after Listing 1, so the reader is first being shown how to call the setsApp commands, before diving into technical details.
2. Listing 1: The code example is incomplete, please define the “serviceRegistrar” variable, how would I get an instance as developer?

**Minor comments and suggestions on the App:** 

1. It doesn't seem possible to compare sets across different networks, the App gives an error when I tried. What is the reasoning behind this? If the networks contain overlapping nodes, wouldn't it be valid to perform the operations across the different networks?
2. In case sets have been defined for multiple networks, it is hard to see in the Sets panel to which network each set belongs. The only way I could find was to click the set and choose “Select” so the nodes get selected in the corresponding network. It would be useful to group the sets by network or indicate the parent network otherwise (i.e. different colors of the red dots).
3. Small GUI tweak proposal: in the dialog where the user needs to specify the set name, it would be handy if the “Enter” key would map to the “Ok” button, so you don’t have to switch to the mouse. It would be more intuitive and speed up the creation of several sets.

I have read this submission. I believe that I have an appropriate level of expertise to confirm that it is of an acceptable scientific standard.

# Peer review: Referee response for version 1

**DOI:** 10.5256/f1000research.4701.r5300

**Published:** 2014-07-14

**Authors:** Jiguang Wang

**Affiliations:** 1 Department of Biomedical Informatics, Columbia University, New York, NY, USA

This manuscript described a new Cytoscape App, named *SetsApp.  *This app provides very simple function about the operations on node or edge sets. It is ****easy to install, and easy to use. I have only minor suggestions for revision:

1. The operation "difference" on three sets is not well defined. This operation should be disabled when selecting three sets.
2. If a new set has the same name with the old one, there will be a warning. This is good, but when I close the warning, my operation is also closed. It will be more convenient if this app goes back to the window of "Enter a new name for the new set".
3. When importing sets from file: if the parameters are not properly set, the app will import nothing. It will be better if warning information tells users the parameters are not properly used.

I have read this submission. I believe that I have an appropriate level of expertise to confirm that it is of an acceptable scientific standard.
